# Ribavirin inhibits the replication of infectious bursal disease virus predominantly through depletion of cellular guanosine pool

**DOI:** 10.3389/fvets.2023.1192583

**Published:** 2023-07-31

**Authors:** Towseef Akram, Irfan Gul, Mahrukh Parveez Zia, Amreena Hassan, Amina Khatun, Riaz Ahmad Shah, Syed Mudasir Ahmad, Nazir Ahmad Ganai, Naveed Anjum Chikan, Won-Il Kim, Nadeem Shabir

**Affiliations:** ^1^Division of Animal Biotechnology, Faculty of Veterinary Sciences and Animal Husbandry, Sher-e- Kashmir University of Agricultural Sciences and Technology of Kashmir, Srinagar, India; ^2^Department of Biotechnology, University of Kashmir, Srinagar, India; ^3^Department of Biotechnology, School of Engineering and Technology, Sharda University, Greater Noida, UP, India; ^4^Faculty of Animal Science and Veterinary Medicine, Sher-e-Bangla Agricultural University, Dhaka, Bangladesh; ^5^Division of Computational Biology, Daskdan Innovations Pvt. Ltd., Srinagar, India; ^6^College of Veterinary Medicine, Jeonbuk National University, Iksan, Republic of Korea

**Keywords:** infectious bursal disease virus, ribavirin, dsRNA, antiviral, mutagen

## Abstract

**Introduction:**

The antiviral activity of different mutagens against single-stranded RNA viruses is well documented; however, their activity on the replication of double-stranded RNA viruses remains unexplored. This study aims to investigate the effect of different antivirals on the replication of a chicken embryo fibroblast-adapted Infectious Bursal Disease virus, FVSKG2. This study further explores the antiviral mechanism utilized by the most effective anti-IBDV agent.

**Methods:**

The cytotoxicity and anti-FVSKG2 activity of different antiviral agents (ribavirin, 5-fluorouracil, 5-azacytidine, and amiloride) were evaluated. The virus was serially passaged in chicken embryo fibroblasts 11 times at sub-cytotoxic concentrations of ribavirin, 5-fluorouracil or amiloride. Further, the possible mutagenic and non-mutagenic mechanisms utilized by the most effective anti-FVSKG2 agent were explored.

**Results and Discussion:**

Ribavirin was the least cytotoxic on chicken embryo fibroblasts, followed by 5-fluorouracil, amiloride and 5-azacytidine. Ribavirin inhibited the replication of FVSKG2 in chicken embryo fibroblasts significantly at concentrations as low as 0.05 mM. The extinction of FVSKG2 was achieved during serial passage of the virus in chicken embryo fibroblasts at ≥0.05 mM ribavirin; however, the emergence of a mutagen-resistant virus was not observed until the eleventh passage. Further, no mutation was observed in 1,898 nucleotides of the FVSKG2 following its five passages in chicken embryo fibroblasts in the presence of 0.025 mM ribavirin. Ribavarin inhibited the FVSKG2 replication in chicken embryo fibroblasts primarily through IMPDH-mediated depletion of the Guanosine Triphosphate pool of cells. However, other mechanisms like ribavirin-mediated cytokine induction or possible inhibition of viral RNA-dependent RNA polymerase through its interaction with the enzyme’s active sites enhance the anti-IBDV effect. Ribavirin inhibits ds- RNA viruses, likely through IMPDH inhibition and not mutagenesis. The inhibitory effect may, however, be augmented by other non-mutagenic mechanisms, like induction of antiviral cytokines in chicken embryo fibroblasts or interaction of ribavirin with the active sites of RNA-dependent RNA polymerase of the virus.

## 1. Introduction

RNA viruses are turning out to be the major causes of global pandemics in recent times. The growing human-animal contact can lead to the evolution of certain viruses and generate diseases with the potential to spread between species and cause pandemics (1, 2). Numerous mutagenic nucleoside analogues have already been approved for use after thorough investigations of their antiviral properties on single-stranded RNA viruses; however, the assessment of these nucleoside analogues against double-stranded RNA viruses is yet to be explored.

Infectious Bursal Disease Virus (IBDV) is a non-enveloped, double-stranded (ds)-RNA virus belonging to the genus Avibirnavirus and family Birnaviridae, that causes a substantial economic impact on the poultry industries globally (3–10). The virus possesses a bi-segmented genome containing segment-A (3.2 kb) that encodes viral proteins; VP2, VP3, VP4, and VP-5 (11–15) and Segment-B (2.8 kb) encoding VP1, which is an RNA-dependent RNA polymerase (RdRp) (16). VP1 contains important active sites necessary for IBDV replication. The active site of the polymerase is formed by Asp-402, Asp-416, and Asn-403. At the same time, Ser-166 amino acid offers a self-guanylylation activity to the virus, which is essential for protein priming, an alternate mechanism of genomic replication of the virus (17).

Ribavirin (1-β-D-ribofuranosyl-2,2,4-triazole-3-carboxamide) is a classic example of base-analogue mutagens clinically used against various viruses affecting human health. Lethal mutagenesis is a primary antiviral mechanism utilized by ribavirin against ssRNA viruses through an accumulation of mutations in the virus beyond a threshold (18–20). However, few studies have explored various non-mutagenic mechanisms utilized by ribavirin to inhibit viral replication (21–27). Although ribavirin has been used clinically as an antiviral for a long time and has been the subject of several studies, the mechanism underlying its antiviral activity is still not entirely understood (28–31). Moreover, previous studies have explored the possible antiviral mechanisms of ribavirin using ssRNA viruses (18, 20, 32–34); however, their effect on the replication of dsRNA viruses remains largely unexplored. The current study employs various strategies to evaluate the inhibitory potential of ribavirin against a dsRNA virus, IBDV, and further explore its possible mechanisms of action using a combination of *in vitro* and comprehensive *in silico* approaches.

## 2. Materials and methods

### 2.1. Viruses and antiviral mutagens

FVSKG2 (Accession number: OP161172), a local IBDV isolate, was used in the study. Four mutagens *viz.*; ribavirin, 5-fluorouracil, 5-azacytidine, and amiloride (Sigma, St. Louis, Missouri, United States) were used in this study. Each mutagen was dissolved separately in Dulbecco’s modified Eagle media (DMEM, Sigma, St. Louis, Missouri, United States) at stock concentrations of 15 mM (Ribavirin), 20 mM (5-fluorouracil, amiloride) and 5 mM (5-azacytidine) which were further sterile-filtered using a 0.22-μm syringe filter (Millex-GV Filter, 0.22 μm Millipore Sigma, Burlington; Massachusetts; United States). The filtrate was aliquoted and stored at −20°C until use, as described in previous studies (35, 36).

### 2.2. Chicken embryo fibroblast cell culture

The chicken embryo fibroblasts (CEFs) were isolated from 9 days old embryonated chicken eggs and maintained in growth media containing DMEM, 5% Fetal Bovine Serum (Fetal Bovine Serum, Sigma, St. Louis, Missouri, United States) and 1% Penicillin–Streptomycin Solution with 10,000 U penicillin and 10,000 μg streptomycin/mL (Pen Strep, Gibco Waltham, Massachusetts, United States), at 37°C and 5% CO2 in a humidified chamber as described in our previous studies (37, 38).

### 2.3. Cytotoxicity assay

The cytotoxic effect of mutagens on CEFs was determined using MTT (3-(4,5-dimethylthiazol-2-yl)-2,5-diphenyltetrazolium bromide) assay (Thiazolyl Blue Tetrazolium Bromide, Sigma, St. Louis, Missouri, United States). Confluent CEFs were prepared in 96-well plates (Tissue culture plate, 96-well Falcon, Flowery Branch, Georgia, United States). Cells were rinsed and replenished with 100 μL growth medium containing one of the four concentrations of mutagens (0, 0.5, 1.0, and 1.5 mM) in three replicates. To determine cell viability at specific time point, cells were incubated at 37°C and 5% CO2, as described in the previous study (35, 39). At 24 hpt and 48 hpt the growth medium of the cells was replaced with 100 μL of DMEM containing MTT at a concentration of 0.5 mg/mL and incubated for 4 h at 37°C. After the MTT treatment, 100 μL of the solubilizing solution containing 10% SDS in 0.01 M HCL was added and the cells were incubated overnight. Absorbance was read at 570 nm using a multimode microplate reader (Biotek, Cytation^™^ 3, Winooski, VT, United States) (40, 41). Control wells (cells with 0.0 mM mutagen) and blank wells (without cells) were utilized for calculating the cell viability by using the following formula as described in our earlier studies (37, 38).


$$\%\mathrm{cell viablity}=\frac{\mathrm{sample}\;\mathrm{abs}.-\mathrm{blank}\;\mathrm{abs}.}{\mathrm{control}\;\mathrm{abs}.-\mathrm{blank}\;\mathrm{abs}.}\times 100$$

### 2.4. Evaluation of the effect of mutagens on IBDV replication

CEFs were cultured in 25 cm^2^ flasks prior to virus inoculation. At the time of virus inoculation, cell number was determined and multiplicity of infection (MOI) of 0.1 was used. The anti-IBDV activity of ribavirin was evaluated against IBDV isolate FVSKG2. Confluent cultures of CEFs were infected with FVSKG2 and incubated for 2 h in a humidified incubator at 37°C and 5% CO2 following which the virus inoculum was discarded, and the cells were replenished with 5 mL maintenance media, each containing a specific concentration of mutagens. Different sub-cytotoxic concentrations of mutagens, ribavirin, 5-Azacytidine, and Amiloride at 0.0, 0.05, 0.1, 0.2, and 0.3 mM, 5-Florouracil at 0.0, 0.1, 0.2, 0.3, and 0.5 mM were tested against FVSKG2 based on previous studies (31, 35, 42, 43). The virus-inoculated cells, each treated with a specific concentration of mutagens, were then incubated for four more days under the same culture conditions as described above, during which time 500 μL of cell culture medium was collected and replaced with fresh media from each flask every 24 h. The collected media was centrifuged and the supernatant was stored at −80°C until further analysis.

### 2.5. Virus titration assay and determination of TCID50

Confluent CEFs in 96-well plates (Tissue culture plate, 96 well, Falcon, Flowery Branch, Georgia, United States) were used to evaluate the titer of FVSKG2 samples. Eight-fold serial dilution (10^–^^1^ to 10^–^^8^) of viruses in DMEM were prepared and inoculated in triplicates with 100 μL media per well and incubated for 2 h under cultural conditions. After incubation, the inoculum was discarded and cells were replenished with growth media. The cells were then incubated for 96 h and monitored for cytopathic effects (CPE). The wells were scored for CPE after 96 h. Virus titer was expressed as 50% tissue culture infective dose (TCID50/mL) and calculated using the method of Reed and Muench (44).

### 2.6. Serial passage of FVSKG2

FVSKG2 was serially passaged in CEFs in different concentrations of mutagens, Ribavirin and Amiloride at 0.0, 0.05, 0.1, 0.2, and 0.3 mM, and 5-Fluorouracil at 0.0, 0.1, 0.2, 0.3, and 0.5 mM for 11 passages. Confluent monolayers of CEFs, prepared in 6-well plates, were pre-treated with mutagens at given concentrations 4hrs prior to inoculation of a virus at 0.01 MOI. After 2 h post incubation, virus inoculum was removed, and cells were replenished with a growth medium containing the same concentrations of mutagens as used in the pre-treatment stage in each well. The infection was then allowed to proceed for 24 h, following which the plates were freeze-thawed thrice. Cell culture fluid was collected from each well and centrifuged at 2,500 rpm for 5 min, and the supernatant was stored at −80°C for virus titration. 200 μL of supernatant from each passage was used as virus inoculum for the next passage. This procedure was repeated for each serial passage of the virus. In addition, the FVSKG2 was serially passaged in the presence of 0.025 mM ribavirin to evaluate for the presence of mutations compared to the control (0 mM Ribavirin).

### 2.7. Viral RNA isolation and sequencing

According to the manufacturer’s instructions (Virus RNA isolation kit, GeneAll, South Korea), 300 μL of the supernatant of the stock virus of FVSKG2 was used to isolate the RNA, with the following changes. The VL buffer was used for 30 min of incubation with the viral sample. The Takara one-step RT-PCR kit was used to perform the one-step RT-PCR (Primescript One-step RT-PCR kit, Takara, Japan). Amplification was performed according to the manufacturer’s instructions, following some modifications suited for amplifying a dsRNA virus. In a 1:4 ratio, DMSO (Sigma Aldrich, ST. Louis, United States) was added to the RNA template with 0.5 μL RNAse inhibitor (rRNasin, Promega, Wisconsin, United States) and incubated at 99°C for 3 min and snap-chilled on ice following which primers, forward A_124F:CGCAGCGATGACAAACCT; reverse A_1100R: GATCCCCCGCCTGACCACCACTT for 976 bp region of segment A and forward B_1080F: CTGAAAGGTACGACAAAAGCACAT; reverse B_2002R: TACCAACCTCAACGCCTCATACCT for 922 bp region in segment B were added separately. Then 12.5 μL of 2X RT-PCR buffer and 1 μL enzyme mix were added to set up a 25 μL reaction in thermo-cycler (Biometra T Advanced, Analytika Jena, Jena, Germany). PCR products of size 976 bp and 922 bp, respectively, were amplified by using the following thermal cycling conditions 50°C for 30 min, 95°C for 2 min, followed by 35 cycles at 95°C for 30 s, 62°C and 61°C, respectively, for 1.5 min, 72°C for 1.5 min, and final extension of 72°C for 5 min. PCR products were purified by DNA purification kit (Wizard^®^ SV Gel and PCR Clean-Up System, Promega Madison, Wisconsin, United States) and sent for sequencing to Macrogen (Seoul, South Korea).

### 2.8. Ribavirin-guanosine inhibition study

To evaluate whether the addition of guanosine may compete with ribavirin and rescue viral replication, FVSKG2 was added at MOI of 0.01 in CEFs. Ribavirin and guanosine combinations were then added to achieve final concentrations of 0, 0.05, 0.05 mM and 0, 0, 0.025 mM, respectively. Culture supernatants were harvested at 48 Hours Post Inoculation (HPI) to determine viral titers as described in previous studies (35, 45).

### 2.9. Inhibition of inosine-5′-monophosphate dehydrogenase

Confluent CEFs in 6-well plates were pre-treated with ribavirin at the concentration of 0, 10 and 40 μM in the presence or absence of a fixed concentration of 40 μM guanosine. Separately, confluent CEFs in 6-well plates were pre-treated with mycophenolic acid at 0, 1, 5, and 10 μM in the presence or absence of fixed concentration of 40 μM guanosine. FVSKG2 at MOI of 0.01 was added to the cells and incubated for 2 h. Inoculum was then removed and the cells were replenished with the same concentrations of the ribavirin, guanosine and mycophenolic acid as in pre-treatment, incubated at 37°C and 5% CO2. The viral titer was determined every 48 h post-infection.

### 2.10. Evaluation of mRNA expression of cytokines

Ribavirin was added at 0, 0.05 and 0.1 mM concentrations in CEFs cultured in 24-well plates. RNA was isolated from the ribavirin-treated CEFs 24-h post-treatment using an RNA isolation kit (GeneAll Hybrid-RTM kit, GeneAll Biotechnology, Seoul, South Korea) following the manufacturer instructions. RNA was reverse-transcribed into complementary DNA (cDNA) using a high-capacity cDNA reverse transcription kit (Thermofisher Scientific, Maasachusetts, United States) as per the following conditions: 25°C for 10 min, 37°C for 120 min and 85°C for 5 min and holding at 4°C. Real-time PCR was performed on Analytik Jena, qRT-PCR (Jena, Germany) system using various cytokine-specific primers (Table 1), following the manufacturer’s instructions. The qPCR reaction was set up using 10 μL of GoTaq qPCR Master Mix(2X), 0.5 μL of Forward Primer (20X), 0.5 μL of Reverse Primer(20X), 7 μL of Nuclease-Free Water, 2 μL of cDNA template (or water for the no-template control reactions) The cycling conditions for performing the qPCR were as follows: GoTaq® Hot Start Polymerase activation for 1 cycle at 95°C for 2 min, denaturation at 95°C for 15 sec, annealing and data collection (40 cycles) at temperature 60°C for 30 sec. The relative quantities of cytokine mRNA in ribavirin-treated and non-treated CEFs were normalized to GAPDH mRNA, and the amounts were determined using the 2-ΔΔCt method (46).

**Table 1:** Primer list for evaluating cytokine expression.

Gene	Primer sequence (5′-3′)	Reference
GAPDH-N-F	CCCCAATGTCTCTGTTGTTG	NM_204305.1
GAPDH-N-R	GCAGCCTTCACTACCCTCTT	
TNF-α-F	CAGATGGGAAGGGAATGAAC	AY765397.1
TNF-α-R	GGTTACAGGAAGGGCAACTC	
IFN-β-F	AATACGGCTCCACCTCCAC	KF741874.1
IFN-β-R	GCTTGCTTCTTGTCCTTGCT	
IL-2-F	TTGGCTGTATTTCGGTAGCA	AF000631.1
IL-2-R	TGGGTCTCAGTTGGTGTGTAG	
IL-6-F	AATCCCTCCTCGCCAATC	HM179640.1
IL-6-R	CCTCACGGTCTTCTCCATAAA	
IL-10-F	TGTCACCGCTTCTTCACCT	NM_001004414.2
IL-10-R	CCCGTTCTCATCCATCTTCT	
IL-12-F	TTTCCTTTGCTGCCCTTCT	AY262752.1
IL-12-R	GGTGTCTCATCGTTCCACTC	
IFN-alpha-F	AACCTTCACCTCACCATCAAA	FJ977575.1
IFN-alpha-R	CGCTGTAATCGTTGTCTTGG	

### 2.11. Protein and ligand structure preparation

Two proteins, RdRp (Uniprot ID: Q9Q6Q5) and IMPDH2 (Uniprot ID: Q5F4A4), and two ligands ribavirin (PubChem ID:37542) and mycophenolic acid (Pubchem ID: 446541) were used in this study. The Crystal Structure of RdRp (PDB ID: 2PGG) (17) was retrieved and used for docking and simulation studies. For IMPDH2, the protein sequence of gallus gallus of 514 residues was obtained from UniProtKB^1^ database. The sequence shared 95% similarity with Human IMPDH2 (PDB ID: P12268), and to generate the three-dimensional structure of IMPDH2 of gallus gallus, the structure representing the IMPDH2 with mycophenolic acid (PDB ID: 1JR1) (47) was used as a template for homology modeling. The modeling was carried out using the SWISS-MODEL server^2^ (48). The generated model was subjected to energy minimization and refinement procedures via the ModRefiner server^3^ (49). The energy-minimized structure was named IMPDH2_GG and was used throughout the study.

### 2.12. Molecular docking

AutoDock Tool (50) was used to perform highly extensive molecular docking. Ribavarin was docked into the crystal structure of RdRp (2PGG), and two separate dockings were performed on two different sites. Site 1 represented the vital amino acids *viz.* ASN402, ASP403, and ASN416 in the catalytic palm of RdRp. Site 2 was built around SER166, a self-guanylation site present in RdRp. For IMPDH2_GG, the potential binding sites were identified based on the structural comparison with human IMPDH2. The crystal structure of IMPDH2 with mycophenolic acid (1JR1) was used as the template to define the binding pocket for IMPDH_GG. Ribavirin and mycophenolic acid were docked into this binding pocket. All the complexes generated were subjected to the molecular dynamics simulation run for further analysis.

### 2.13. All atom molecular dynamics simulation

GROMACS 2021 series version-2 (51) molecular dynamics package was used to carry out an all-atom molecular dynamics simulation of the four complexes generated from molecular docking. The CHARMM36 (52) force field was used to define the complex of protein, water, and ions in the TIP3P water model. The ligands used, *viz.* ribavirin, and mycophenolic acid were processed in the CHARMM General Force Field (CGenFF) program^4^ (53). Energy Minimization and conjugate gradient algorithms of GROMACS were employed for optimizing the final protein-ligand complexes. The protein, ligand, water, and ions systems were equilibrated in NVT and NPT ensembles for 100 ps. Using the Parrinello–Rahman barostat, these ensembles maintained the system at 310 K temperature and 1 bar of pressure. Each production run included three replicas of 100 ns. Trajectories were saved every 2 ps/frame for further analysis.

### 2.14. Data analysis

The effect of mutagens on CEFs was analysed by Mann–Whitney U test while the effect of mutagen on FVSKG2 replication, serial passages and multi-step growth curve was analyzed by Repeated measures analysis of variance (ANOVA) followed by Dunnet’s post-test using GraphPad Prism 8 (DNASTAR (Inc., Madison, WI, United States). Nucleotide sequences were aligned and analyzed using DNASTAR (Inc., Madison, WI, United States). Cytokine expression was analyzed by the Mann–Whitney U test (SPSS Version 20) (35, 54). This study performed the primary docking using AutoDock Vina (The Scripps Research Institute, United States) and molecular dynamics simulation using the GROMACS-2021. Discovery Studio Biovia 2021 (Dassault Systèmes, USA) and PyMOL (Schrödinger, LLC) were employed for visualizations.

## 3. Results

### 3.1. Ribavirin exhibited the least cytotoxicity on CEFs

**Figure 1:**
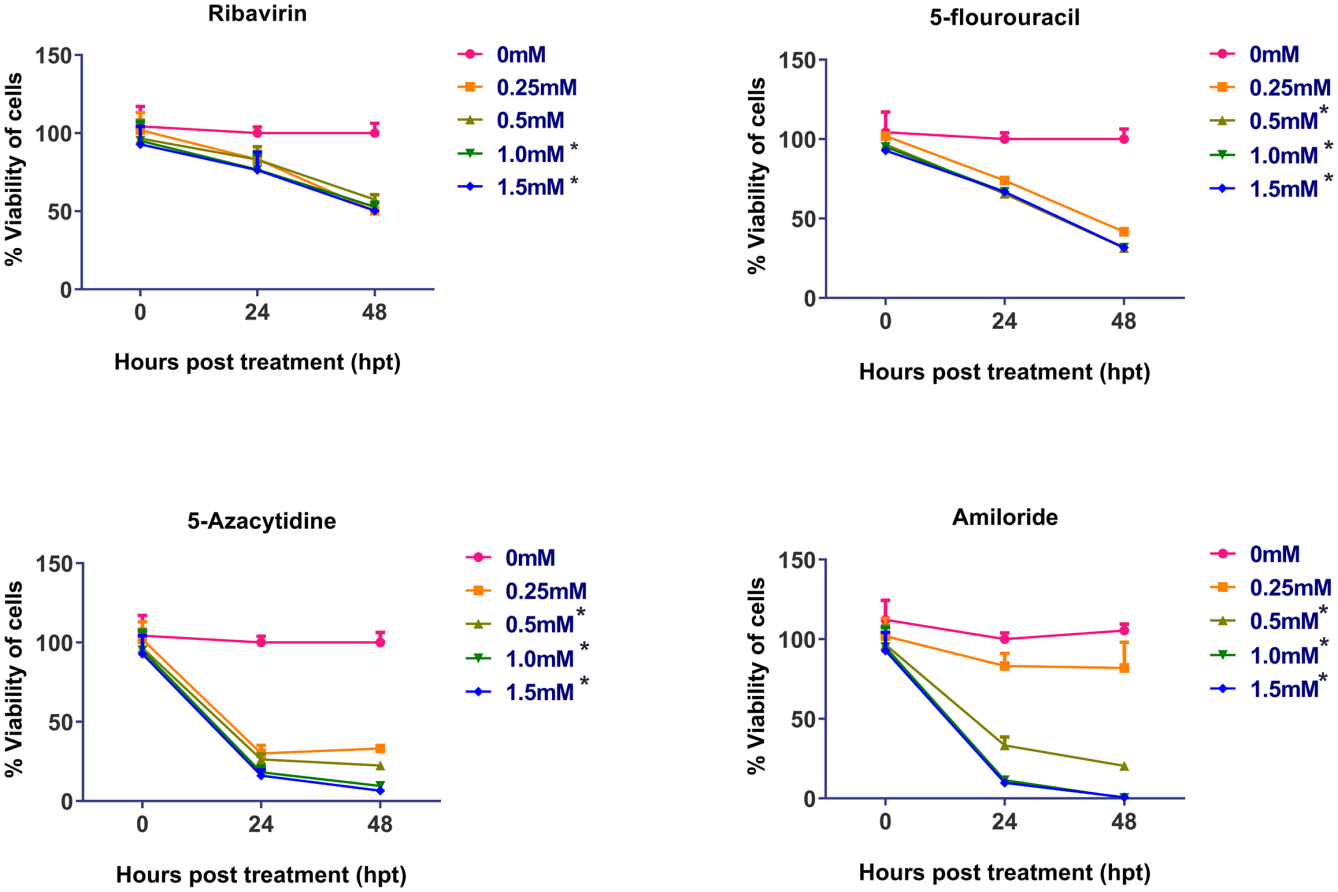
Cytotoxicity assay of mutagens on CEFs: Cell viability was determined by using MTT assay: Y-axis represents percent cell viability, while X-axis represents hours post-treatment (hpt). Data expressed as mean ± standard error (*n* = 3). Significant cytotoxicity was observed at and above 1.0 mM of ribavirin, while as in case of 5-fluorouracil, 5-azacytidine and amiloride significant cytotoxicity was observed at 0.5 mM. Asterisks represent significant difference (*p* < 0.05). The number of asterisks “*” represents the extent of significance. The 0 hpt refers to time period immediately after the treatment.

The cytotoxicity of the four mutagens was evaluated and is summarized in the Figure 1. No significant cytotoxicity was observed after 48 h post-treatment at concentration of 0.25 mM and 0.5 mM, but at 1.0 and 1.5 mM, ribavirin was found to be significantly cytotoxic. 5-fluorouracil, 5-azacytidine and amiloride showed significant cytotoxicity at 0.5 mM concentration and above.

### 3.2. Ribavirin inhibited the IBDV replication in CEFs in a dose-dependent manner

**Figure 2:**
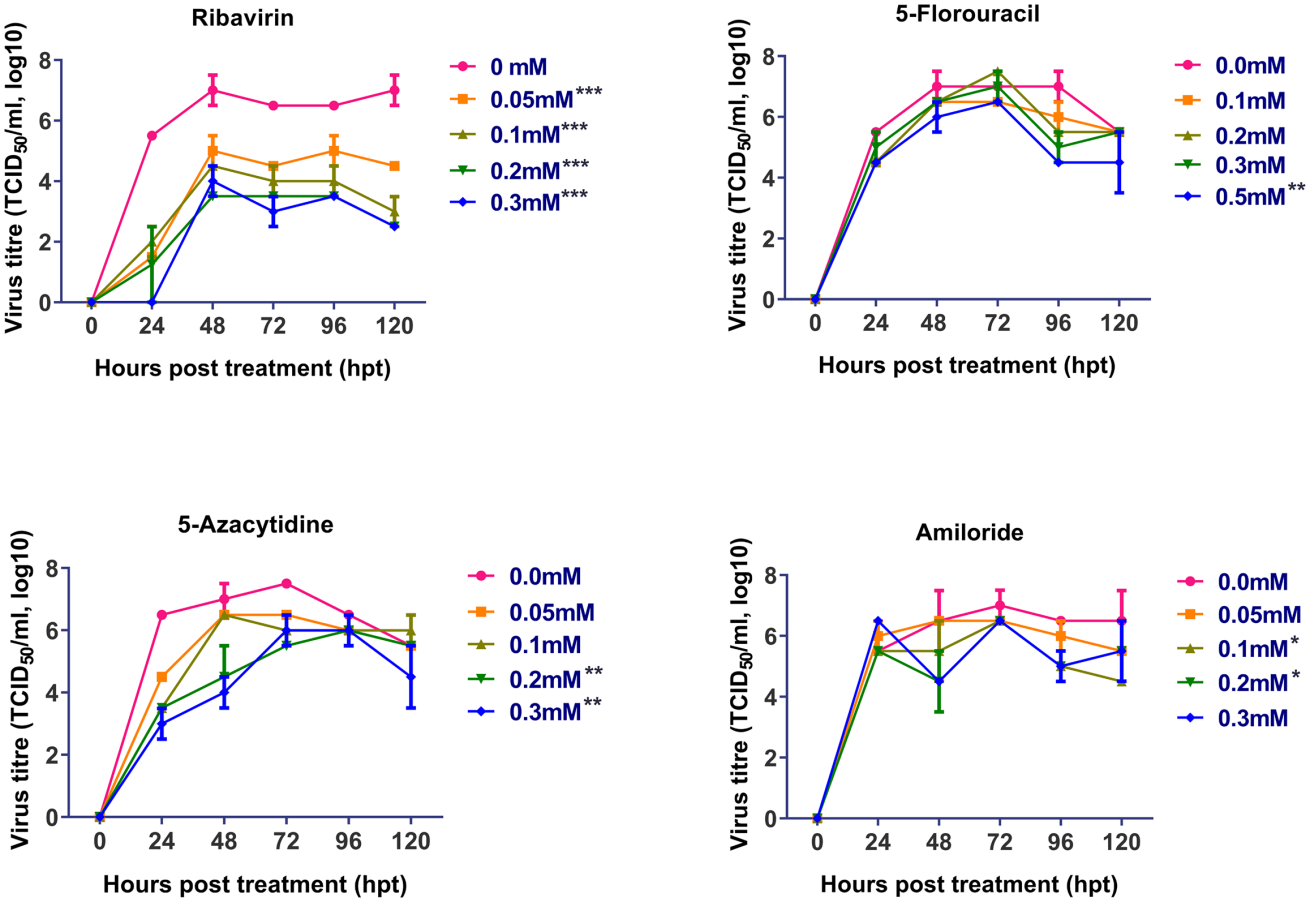
Effect of ribavirin, 5- fluorouracil, 5-azacytidine, and amiloride on replication of FVSKG2: The effect of indicated concentrations of the mutagens on the replication of IBDV isolate FVSKG2 in CEFs were evaluated. Results are presented as 50% tissue culture infective dose TCID50 per mL (y-axis) over time (x-axis). Virus titers were evaluated using cell culture fluids collected from pre-treated CEFs every 24 h after being incubated with the FVSKG2. At 0.05, 0.1, 0.2, and 0.3 mM of ribavirin, the replication of FVSKG2 significantly decreased in a dose-dependent manner. At 0.5 mM of 5-fluorouracil, a 2 log_10_ drop in virus titer was seen along with significant suppression of FVSKG2 replication (*p* = 0.0001). On the other hand, there was no detectable antiviral action with 0.1, 0.2, and 0.3 mM 5-fluorouracil. Treatment with 5-azacytidine at 0.2 and 0.3 mM significantly reduced FVSKG2 replication by up to 2 log_10_ (*p* = 0.0019). Similarly, significant inhibition of FVSKG2 replication was caused by high concentrations (1 and 2 mM) of amiloride. Error bars denote mean ± standard error of the mean (SEM) while Asterisks “*” indicate significant differences in virus titer (TCID50/mL log_10_) as compared to control (0 mM), *p* < 0.05. The number of asterisks represents the extent of significance.

The antiviral effect of ribavirin, 5- fluorouracil, 5-azacytidine and amiloride on replication of FVSKG2 in CEFs was evaluated using a multi-step growth curve. This was done by determining the titer (TCID50/mL) of mutagen-treated viral populations at indicated concentrations relative to the control concentration (0 mM) of mutagens, summarized in Figure 2. The replication of FVSKG2 decreased significantly at 0.05, 0.1, 0.2, and 0.3 mM of ribavirin in a dose-dependent manner (*p* < 0.0001). When FVSKG2 was treated with 0.3 mM ribavirin, there was about a 3.5-log_10_ reduction in virus titer at 72 h post treatment, followed by a 4.5-log_10_ reduction at 120 hpt. Although similar activity was observed with 0.2 mM ribavirin, a 4-log_10_ reduction and 2.5-log_10_ reduction were observed in the presence of 0.1 mM or 0.05 mM ribavirin, respectively. Significant suppression of FVSKG2 replication was observed at 0.5 mM of 5-fluorouracil with 2-log_10_ reduction in virus titer (*p* < 0.0001). However, no significant antiviral activity was observed with 0.1, 0.2, and 0.3 mM 5-fluorouracil. Treatment with 0.2 and 0.3 mM 5-azacytidine significantly achieved up to 2-log_10_ reduction in FVSKG2 replication (*p* = 0.0019). Similarly, high concentrations (1 and 2 mM) of amiloride also caused significant suppression of FVSKG2 replication with up to 2.5-log_10_ reduction in virus titer (*p* = 0.0266). Further, no significant antiviral activity was measured at low concentrations of 5-azacytidine (<0.1 mM) and amiloride (<0.05 mM).

### 3.3. Ribavirin drove an early extinction of IBDV in CEFs during serial passages

**Figure 3:**
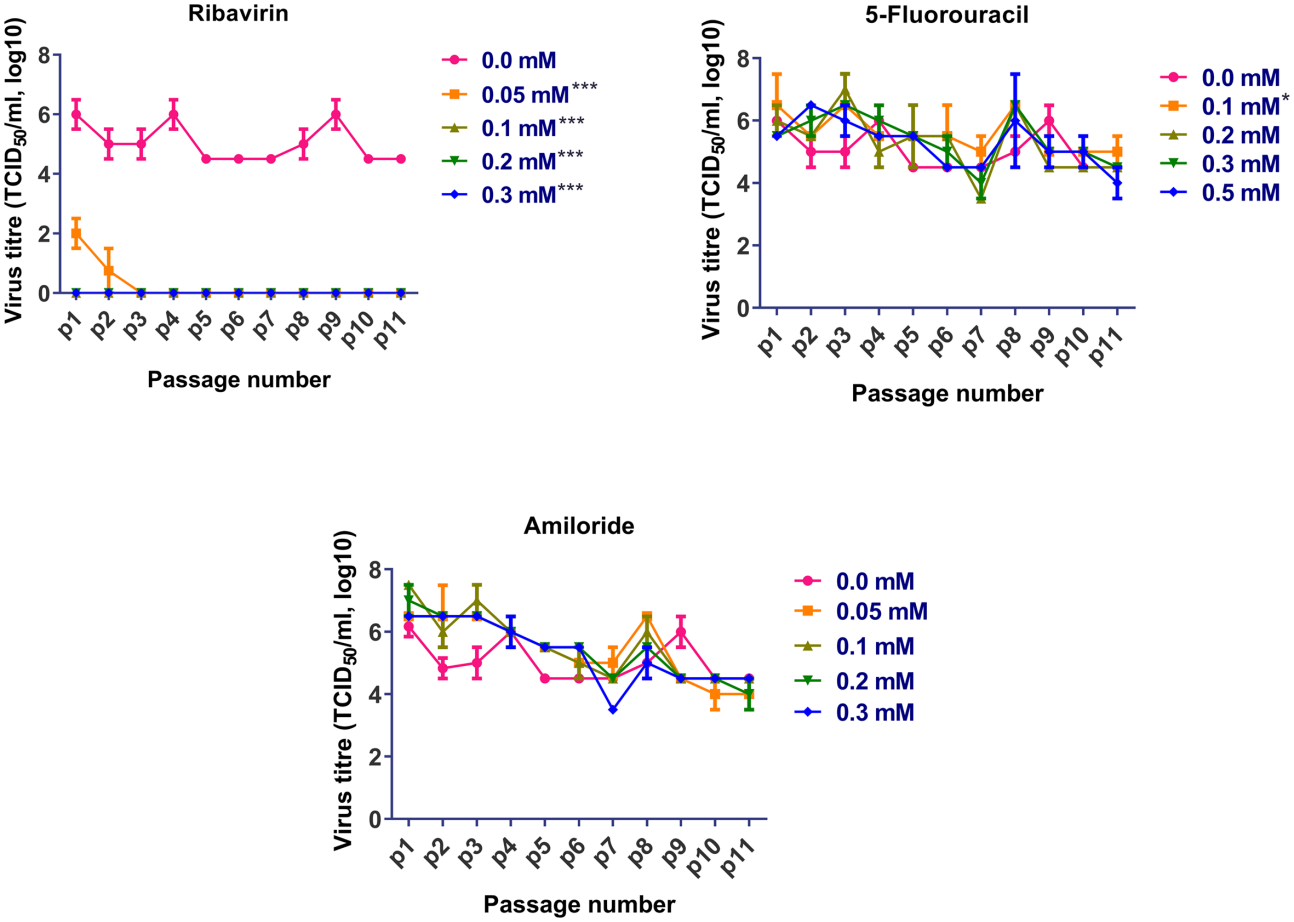
Sequential passage of FVSKG2 in different concentrations of ribavirin, 5-fluorouracil, and amiloride in CEFs: In presence of ribavirin at concentrations of 0.05 and above 0.1 mM, extinction of virus occurred during third and first passage, respectively. In presence of 5-fluorouracil no significant inhibition of FVSKG2 was observed at concentrations at and above 0.1 mM in CEFs. In presence of amiloride, no significant inhibition of FVSKG2 was observed at concentration at and above 0.05 mM in CEFs, Asterisks “*” indicate significant differences.

Based on the cytotoxicity and antiviral activity of the selected antiviral mutagens, serial passages of FVSKG2 in CEF cells were carried out in the presence of an indicated concentration of each mutagen (Figure 3). We observed that among the mutagens, ribavirin was active against FVSKG2, reducing the viral titer in a concentration-dependent manner. When passaged in the presence of 0.05 mM ribavirin, virus titers dropped for the first two passages causing a significant 4-log_10_ reduction at p1 and p2. However, the virus disappeared in the third passage and did not emerge until the eleventh passage. Furthermore, FVSKG2 replication was completely suppressed at higher ribavirin concentrations (>0.1 mM). The virus appeared to be less sensitive to 5-fluorouracil and amiloride at indicated concentrations, with no significant change in the virus titer across the 11 passages relative to the control (0 mM). The only exception was the 0.1 mM 5-fluorouracil, which exhibited significant fluctuations in virus titers (*p* < 0.05). In the presence of 5-fluorouracil at a concentration of 0.1 mM, the virus initially experienced a 1-log_10_ reduction in titer at passage 2, reaching an equilibrium from passage 4 to passage 6 with 10^5.5^ TCID50/mL virus titer. By passage 8, the virus titer increased again to 10^5.5^ TCID50/mL and reached a new equilibrium by passage 9 with a 1.5 log_10_ reduction in virus titer. The virus titer persisted at 10^5^ TCID50/mL from passage 9 to passage 11.

### 3.4. Ribavirin did not induce any mutations in FVSKG2 at 0.025 mM

FVSKG2 replication was inhibited to an undetectable level at 0.05 mM of ribavirin in CEFs. However, at 0.025 mM ribavirin, FVSKG2 was replicated at a low titer. No mutation was observed in 1,898 nucleotide bases in FVSKG2 passaged five times in the presence of 0.025 mM ribavirin in CEFs when compared to FVSKG2 passaged for the same number of times in the absence of ribavirin.

### 3.5. Ribavirin competition assay with guanosine

**Figure 4:**
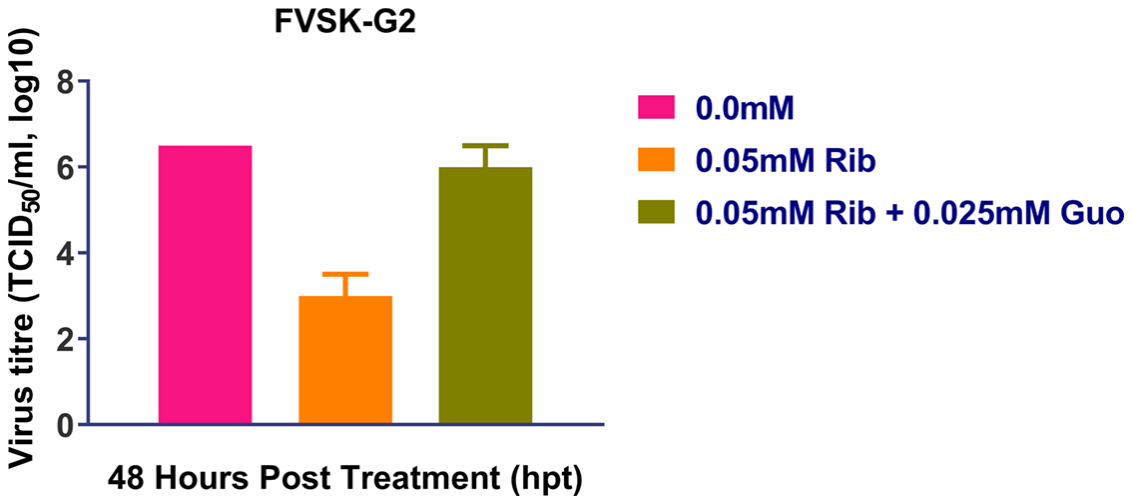
Ribavirin competition assay with guanosine: Ribavirin (Rib) competition assay with nucleoside guanosine (Guo) was performed with FVSKG2 at MOI of 0.01 in CEFs, under three different conditions: no drug treatment, 0.05 mM of ribavirin and 0.05 mM ribavirin plus 0.025 mM guanosine. Culture supernatants were harvested at 48 h post-treatment to determine viral titers. The mean viral titers ± s.d. from triplicates derived from one out of two independent experiments are shown.

At 48 h after treatment, ribavirin inhibited the replication of FVSKG2, resulting in a viral titer of 10^2^ TCID50/mL and a 5.5-log_10_ reduction in viral titer compared to 0 mM ribavirin. However, when 0.025 mM guanosine was added in addition to ribavirin, the viral titer increased to 10^6^ TCID50/mL at 48 h. Therefore, Ribavirin inhibited FVSKG2 replication, while adding guanosine to the culture revived the virus replication (Figure 4).

### 3.6. Inhibition of IMPDH by ribavirin

**Figure 5:**
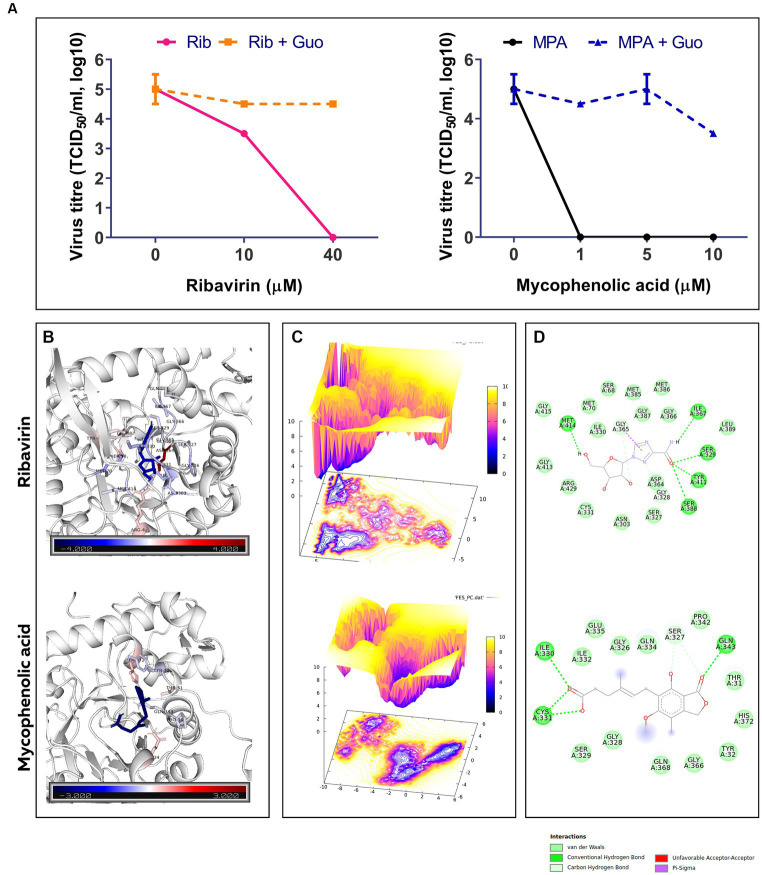
**(A)** The effect of indicated concentrations of ribavirin (Rib) and mycophenolic acid (MPA) in presence or absence of Guanosine (Guo) Supplementation on the replication of FVSKG2 in CEFs. Virus titers are presented as 50% tissue culture infective dose (TCID50/mL, log_10_) per milliliter (y axis) over drug concentration (x axis). Errors bars denote mean ± standard error of mean (SEM). **(B)** B-factor diagram indicating binding free-energy contribution of interacting residues. **(C)** FEL values constructed as a function of PC1 and PC2 eigenvectors. **(D)** The ligand-IMPDH interaction diagram showing the presence of important interactions.

The replication of FVSKG2 in CEFs was investigated at indicated concentrations of ribavirin and mycophenolic acid in the presence or absence of 40 mM guanosine. In the non-guanosine treated control, low titers of FVSKG2 have measured at 10 uM ribavirin, reaching a peak titer of almost 10^3.5^ TCID50/mL at 48 h post-infection. However, no virus was detected at 40 μm ribavirin in cell-free supernatant. Guanosine addition increased the titers significantly, with titers reaching >10^3.5^ TCID50/mL at 48 h post-infection. At 10 μm ribavirin, guanosine addition caused a 1-log_10_ increase in virus titer and completely rescued the virus at 40 μm ribavirin with titer reaching 10^4.5^ TCID50/mL. Consistent with this, similar findings were observed in case of mycophenolic acid, wherein guanosine supplementation repressed the antiviral effect of the mycophenolic acid. Intriguingly, mycophenolic acid inhibited the FVSKG2 replication at the indicated concentration (>1 μM). The addition of guanosine resulted in a 4.5 log_10_ increase in virus titer at 1 μM mycophenolic acid, which persisted to higher concentrations of mycophenolic acid. The virus achieved a 3.5-log_10_ increase in titer at 10 μM mycophenolic acid, reaching about 10^3.5^ TCID50/mL at 48 h post-infection compared to non-guanosine treated mycophenolic acid control (Figure 5A).

Molecular docking of ribavirin and mycophenolic Acid with IMPDH was performed using Autodock vina. As a potent IMPDH inhibitor, mycophenolic acid was chosen as the standard reference molecule. Analysis of the molecular interactions summarized in Table 2 showed negative binding energy for both molecules within the IMPDH binding pocket. While Mycophenolic Acid (MPA) exhibited a binding energy of −5.9 Kcal/mol, ribavirin with IMPDH exhibited a binding energy of −8.1 Kcal/mol. The molecular interactions revealed the presence of Van der wall interactions as well as hydrogen bonding, indicating efficient ligand binding (Figure 5D). Overall, these studies suggested stable binding of ribavirin compared to the reference molecule within the IMPDH binding pocket (S1 Movie). Ribavirin can thereby prevent viral replication by acting as a potent inhibitor of IMPDH.

**Table 2:** Molecular Docking Presentation of binding affinity and binding pockets of Mycophenolic Acid and Ribavirin with IMPDH.

Compounds	Binding affinity (Kcal/mol)	Binding pockets
Mycophenolic acid	−5.9	ILE:330, GLY:326, SER:327, ILE:332, GLN:334, GLU:335, PRO:342, GLN:343, THR:31, HIS:372, TYR:32, GLY:366, GLN:368, GLY:328, SER:329, CYS:331
Ribavirin	−8.1	GLY:415, MET:414, MET:70, SER:68, MET:385, MET:386, ILE:330, GLY:387, GLY:366, ILE:367, LEU:389, SER:329, TYR:411, SER:388, ASP:364, GLY:328, SER:327, ASN:303, CYS:331, ARG:429, GLY:413, GLY:365

Amino acids are represented as three capital letter symbols.

The docked complexes were subjected to molecular dynamics simulation and MMPBSA analysis using GROMACS and gmx_MMPBSA, respectively. The residual decomposition energy of the interacting residues was calculated at an interval of 20 ps, representing 500 frames over the 100 ns trajectories. The per residue energy decomposition plot showed stable and persistent binding of ribavirin to IMPDH compared to mycophenolic acid (Supplementary Figure S1). In addition, the energy2bfac tool used to map the binding energy contribution of interacting residues illustrated the involvement of a larger number of residues in the binding of ribavirin within the IMPDH binding pocket (Figure 5B). The Free Energy Landscape (FEL) values of Rib-IMPDH and MPA-IMPDH were constructed and plotted (Figure 5C). A comparison of the FEL values for the complexes revealed that Rib-IMPDH FEL spanned significantly larger areas of PC1 and PC2 with more free energy wells in the region of the global free energy minimum region. These findings indicate greater flexibility and conformational diversity of Rib-IMPDH compared to the MPA-IMPDH complex.

### 3.7. Effect of ribavirin on the expression of cytokines in chicken embryo fibroblasts

**Figure 6:**
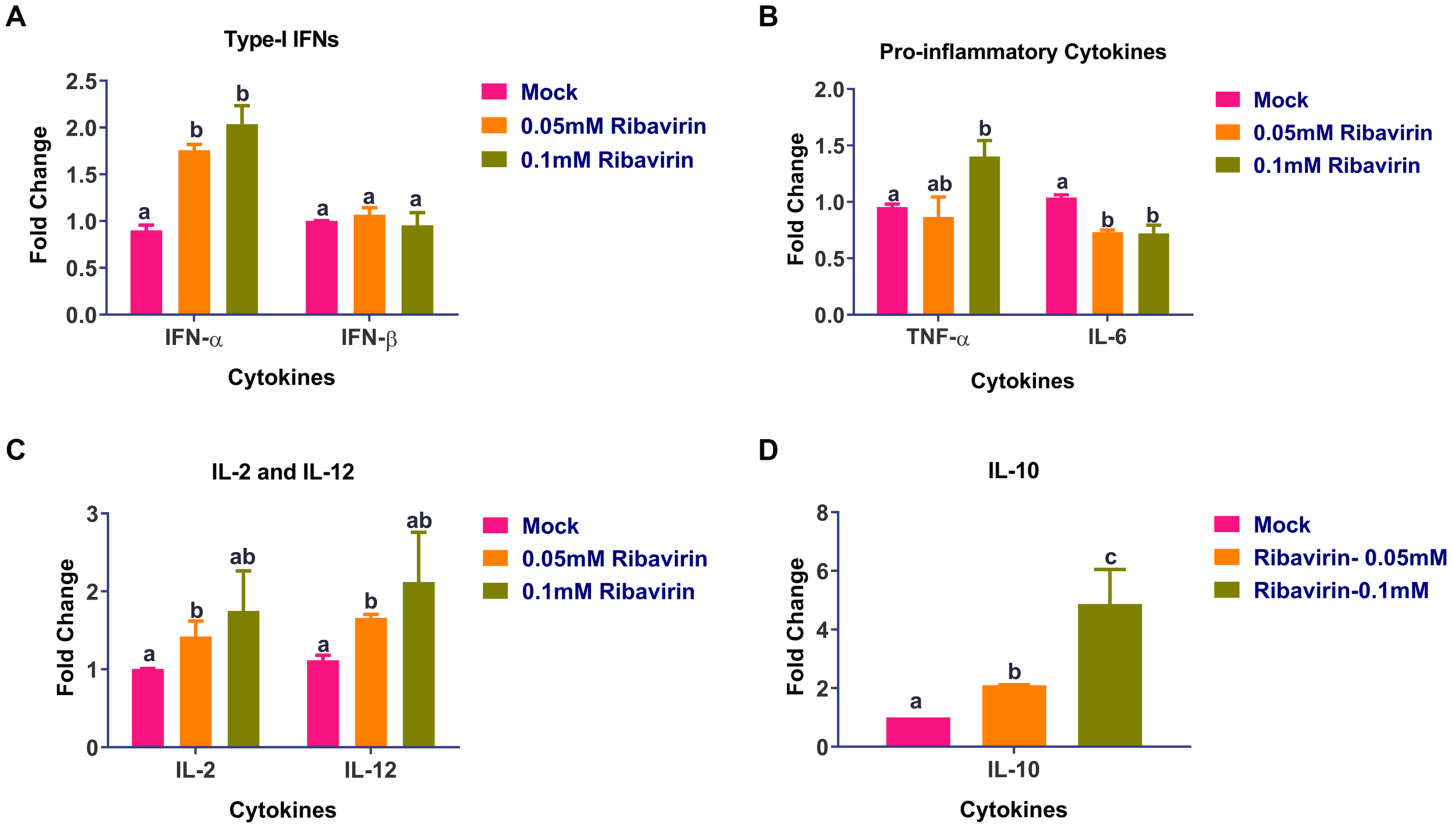
Evaluation of cytokines alteration in CEF cells by ribavirin: CEFs were grown in the presence or absence of indicated concentrations of ribavirin. CEFs were harvested at 24 hpt and subjected to cytokine mRNA expression analysis. Cytokines expression analysis of IL-6, IL-2, IL-12, interferon-alpha (IFN-α), tumor necrosis factor-alpha (TNF-α) and TNF-β mRNAs in CEF cells after 24-h culture. qRT-PCR was used for evaluating mRNA expression. While Relative quantification (RQ) was calculated using the 2-DDCt method. Error bars denote mean ± standard error of the mean (SEM). The bars represent the means, and the error bars represent the standard errors of the mean (SEM). Bars showing different letters represent values that differ significantly from each other (*p* < 0.05).

Real-time PCR was used to assess the cytokine levels in CEFs following a 24-h treatment with 0.05 or 0.1 mM ribavirin. As compared to mock-treated CEFs, the expression of IFN-α (Figure 6A), TNF-α (Figure 6B), IL-2, IL-12 (Figure 6C), and IL-10 (Figure 6D) was significantly higher in CEFs treated either with 0.05 or 0.1 mM ribavirin. However, there was no significant difference in the expression of IFN-β between mock-treated and ribavirin-treated CEFs. Further, the expression of IL-6 in CEFs treated with 0.05 or 0.1 mM of ribavirin was significantly lower than in the mock-treated CEFs (Figure 6B).

### 3.8. *In silico* analysis of ribavirin as inhibitor of RdRp of IBDV

**Figure 7:**
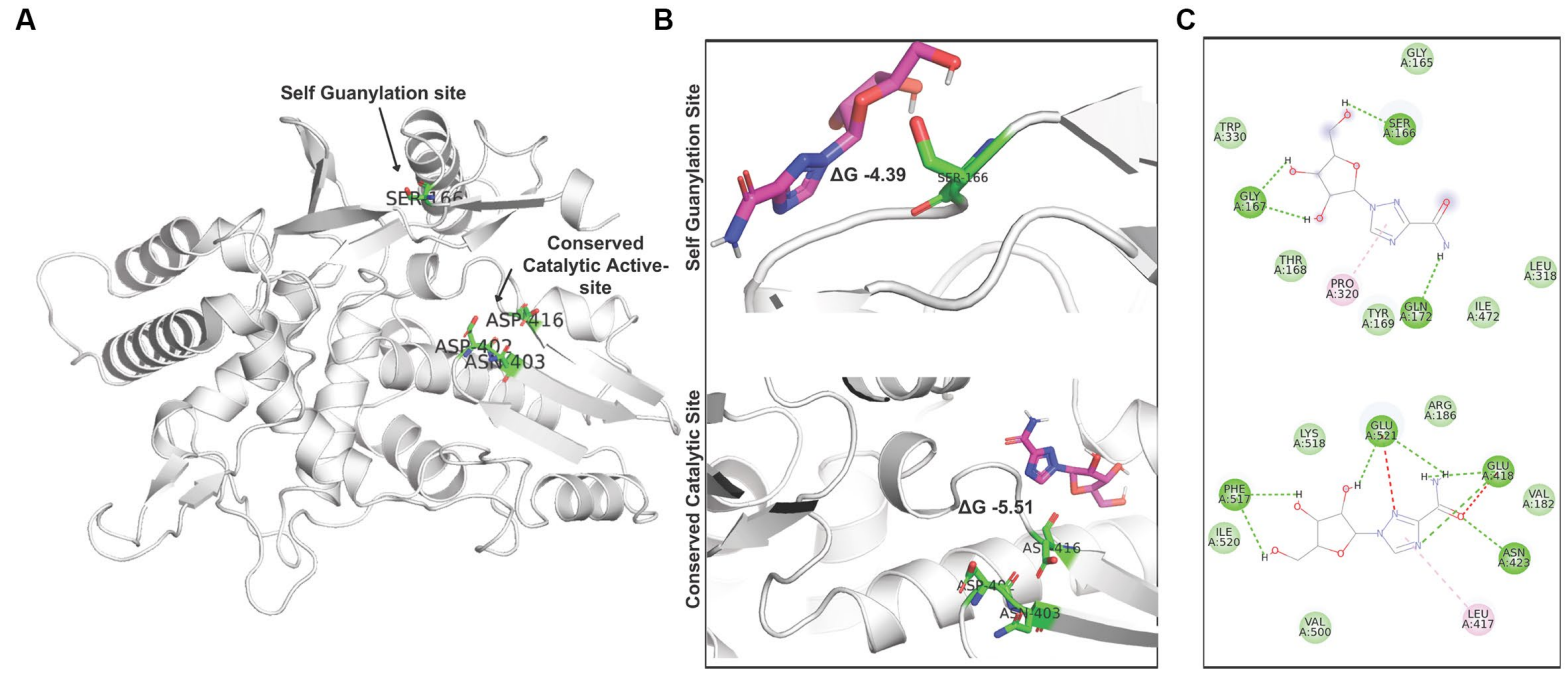
Interaction of ribavirin with RdRp: **(A)** Denotion of the target sites in the IBDV RdRp structure. **(B)** Autodock vina based Molecular docking analysis of ribavirin at site 1 and site 2. **(C)** Analysis of interacting residues and nature of interaction between ribavirin and IBDV RdRp target sites.

Ribavirin shows a brief interaction with the two active sites of RdRp (S2 Movie). While site-1, formed of ASP 402, ASN 403, and ASP 416, constitutes the conserved catalytic active site, site-2, formed by SER 166, constitutes the RdRp self-guanylation site (Figure 7A). Ribavirin shows decent binding energy within both target sites. The analysis of the docking results revealed binding energy of −4.39 kcal/mol at site 2 and − 5.51 kcal/mol at site-1 (Figure 7B). The presence of Van der wall interactions and hydrogen bonding indicate efficient binding of ribavirin at both the target sites (Figure 7C).

## 4. Discussion

In the current study, ribavirin was found to be the least cytotoxic on CEFs among the tested mutagens, which agrees with the previous studies that used different cells (18, 35, 55). Ribavirin significantly inhibited the replication of FVSKG2 at concentrations of 0.05 mM and above which is in agreement with previous studies where the antiviral activity of ribavirin has been demonstrated against many other RNA viruses like influenza virus, poliovirus, human respiratory syncytial virus, hantaan virus and foot and mouth disease virus (FMDV) (18, 20, 32–34).

The mechanism of action of ribavirin on viral replication is not fully understood despite its use as an antiviral (28–30). In the current study, the treatment of FVSKG2 with 0.025 mM ribavirin for series of five passages could not induce any mutations in the 1,898 base pairs of viral genome sequenced, which is in disagreement with previous studies where antiviral activity of ribavirin on ssRNA viruses, *viz.* FMDV, HCV, polio virus, hepatitis E virus and mumps virus was found to be due to mutagenic action of ribavirin (20, 32, 39, 56, 57). The reasons for no-mutagenic action of ribavirin on FVSKG2 may be due to variable reasons: (a) dsRNA are reported to be less prone to mutations than ssRNA viruses (58), (b) Sequencing only a part of viral genome, and (c) using sanger’s sequencing method which cannot detect the sub-consensus variants that could have likely generated during the treatment of virus with 0.025 mM ribavirin.

In the current study, ribavirin inhibited IBDV replication through guanosine depletion which is in agreement with previous studies where ribavirin was found to deplete guanosine pool of cells (18). Further, in the current study, the ribavirin-mediated depletion of the guanosine pool of cells was demonstrated to occur due to inhibition of IMPDH by ribavirin (Figure 5), which was further validated by the strong interaction of ribavirin with IMPDH shown by the *in-silico* studies. This is in agreement with previous studies where the antiviral effect of ribavirin on flaviviruses, paramyxoviruses, dengue virus and influenza A virus was found to be due to depletion of cellular guanosine pool via IMPDH inhibition (26, 59–62).

Ribavirin showed brief interaction with two active sites of RdRp of IBDV in the current study (Figure 6) which is in agreement with previous studies where ribavirin was observed to possess a weak inhibitory activity on RdRp of many RNA viruses *viz.* HCV, Hepatitis E virus, bovine viral diarrhea virus, vesicular stomatitis virus, influenza virus, reovirus, and HIV *in vitro* or *in silico* (23, 24, 63).

We observed a significantly higher mRNA expression of IFN-α, TNF- α, IL-2, IL-12, and IL-10 was in CEFs treated with 0.05 or 0.1 mM ribavirin which is partly in agreement with previous studies where ribavirin was also found to up-regulate the interferon-stimulated genes and enhance the effect of interferons (21, 22). In another study, ribavirin was found to augment IL-2, IFN-α, and TNF-α and suppress IL-4 and IL-5 (24). Further, the expression of IL-6 in CEFs treated with 0.05 or 0.1 mM of ribavirin was significantly lower than in mock-treated CEFs which is in agreement with a study where ribavirin was shown to likely suppress IL-6 in alveolar epithelial cells (64). Collectively, ribavirin inhibited the replication of FVSKG2 predominantly through the ribavirin-mediated guanosine depletion of CEFs; however, other mechanisms like induction of antiviral cytokines in CEFs by ribavirin or the interaction of ribavirin with the active sites of viral RdRP could also have an additive effect on the inhibition of the virus replication.

## 5. Conclusion

The study reveals that dsRNA viruses are likely more resistant to ribavirin-mediated mutagenesis compared to the ssRNA viruses. Ribavirin inhibits dsRNA viruses through non-mutagenic mechanisms, primarily through depletion of the guanosine pool of cells which is likely augmented by its stimulation of cellular cytokines or its inhibition of active sites of viral RdRp.

## Data availability statement

The data presented in the study are deposited in the NCBI repository (https://www.ncbi.nlm.nih.gov/), accession number OP161172.1.

## Ethics statement

The animal study was reviewed and approved by Institutional Animal Ethics Committee (IAEC), Faculty of Veterinary Sciences and Animal Husbandry, Sher-e-Kashmir University of Agricultural Sciences and Technology of Kashmir (Registration no: 1809/GO/ReBi/15/CPCSEA).

## Author contributions

TA and MP performed the in vitro experiments and drafted the manuscript. IG performed the part of *in-vitro* and *in silico* study and drafted the manuscript. W-IK and AK conducted thorough reviews and editing of the manuscript. AH performed additional *in vitro* experiments suggested by the reviewers. RAS, SMA, and NAG contributed to the reading and revision of the manuscript. NS and NC contributed to the conception and design of the study, the data analyses, and manuscript revisions. All authors contributed to the article and approved the submitted version.

## Funding

This work was supported by a grant from the Science and Engineering Research Board, Department of Science and Technology, Government of India, under the early career research award scheme (No: ECR/2016/001172).

## Conflict of interest

NC is employed by Daskdan Innovations Pvt. Ltd.

The remaining authors declare that the research was conducted in the absence of any commercial or financial relationships that could be construed as a potential conflict of interest.

## Publisher’s note

All claims expressed in this article are solely those of the authors and do not necessarily represent those of their affiliated organizations, or those of the publisher, the editors and the reviewers. Any product that may be evaluated in this article, or claim that may be made by its manufacturer, is not guaranteed or endorsed by the publisher.

## Footnotes

^1^http://www.uniprot.org/

^2^https://swissmodel.expasy.org/

^3^https://zhanggroup.org/ModRefiner/

^4^https://cgenff.umaryland.edu/
