# Supplementary material for: Ribavirin inhibits the replication of infectious bursal disease virus predominantly through depletion of cellular guanosine pool
Source: Front Vet Sci. 2023 Jul 31;10:1192583. doi: 10.3389/fvets.2023.1192583 (PMC10433155; doi:10.3389/fvets.2023.1192583)
Supplement: Supplementary file 3 [file Data_Sheet_1.docx]

Supplementary Material

**Ribavirin inhibits the replication of infectious bursal disease virus predominantly through depletion of cellular guanosine pool**

Towseef Akram^1^, Irfan Gul^1,2^, Mahrukh Parveez^1^, Amreena Hassan^1,2^, Amina Khatun^3^, Riaz Ahmad Shah^1^, Syed Mudasir Ahmad^1^, Nazir Ahmad Ganai^1^, Naveed Anjum Chikan^4^, Won-Il Kim^5*^, Nadeem Shabir^1*^.

^1^Division of Animal Biotechnology, Faculty of Veterinary Sciences and Animal Husbandry, Shuhama, Sher-e- Kashmir University of Agricultural Sciences and Technology of Kashmir, Srinagar, India.

^2^Department of Biotechnology, University of Kashmir, Srinagar, India.

^3^ Faculty of Animal Science and Veterinary Medicine, Sher-e-Bangla Agricultural University, Dhaka, Bangladesh.

^4^Division of Computational Biology, Daskdan Innovations Pvt. Ltd. Kashmir, Srinagar, India.

^5^College of Veterinary Medicine, Jeonbuk National University, 79 Gobong-ro, Iksan, 54596,
South Korea.

***Correspondence:**

**Corresponding Author:**

Dr. Nadeem Shabir,

Division of Animal Biotechnology, Faculty of Veterinary Sciences and Animal Husbandry, Shuhama, Sher-e- Kashmir University of Agricultural Sciences and Technology, Kashmir, 190006, India. Email: [nadeem.shabir@skuastkashmir.ac.in](mailto:nadeem.shabir@skuastkashmir.ac.in)

**Co-Corresponding Author:**

Dr. Won-Il Kim

College of Veterinary Medicine, Jeonbuk National University, 79 Gobong-ro, Iksan, 54596,
South Korea. Email: [kwi0621@jbnu.ac.kr](mailto:kwi0621@jbnu.ac.kr)

# Supplementary Videos

S1 Video: In Silico binding analysis of Ribavirin and Mycophenolic acid with IMPDH

S2 Video: In Silico binding analysis of Ribavirin at two target sites of IBDV RdRp

## Supplementary Figures

##
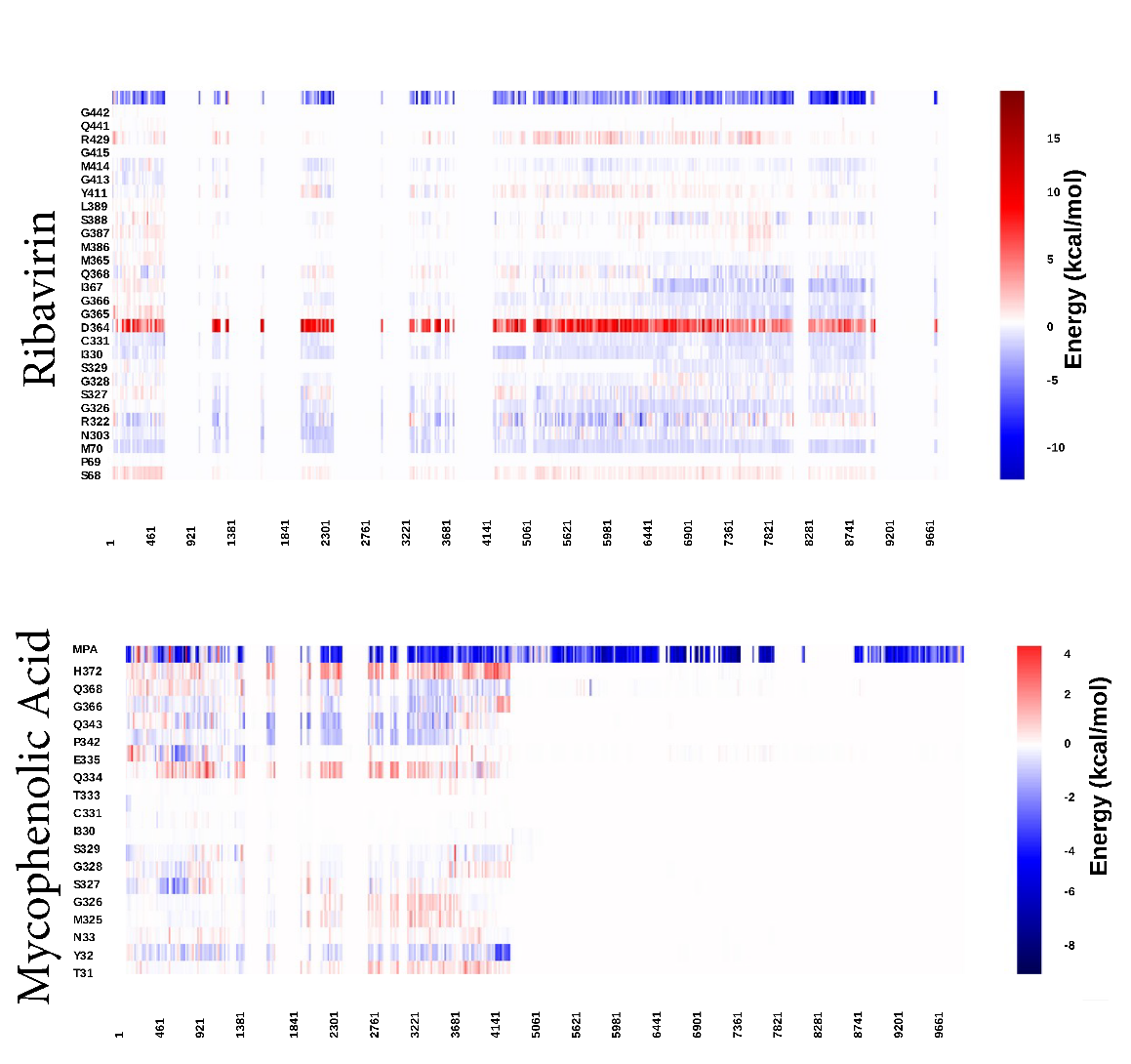


## Fig S1. MM-PBSA per-residue decomposed energy of IMPDH with Ribavirin and Mycophenolic Acid. *Colour bar represents the relative free-energy values in kcal/mol.*


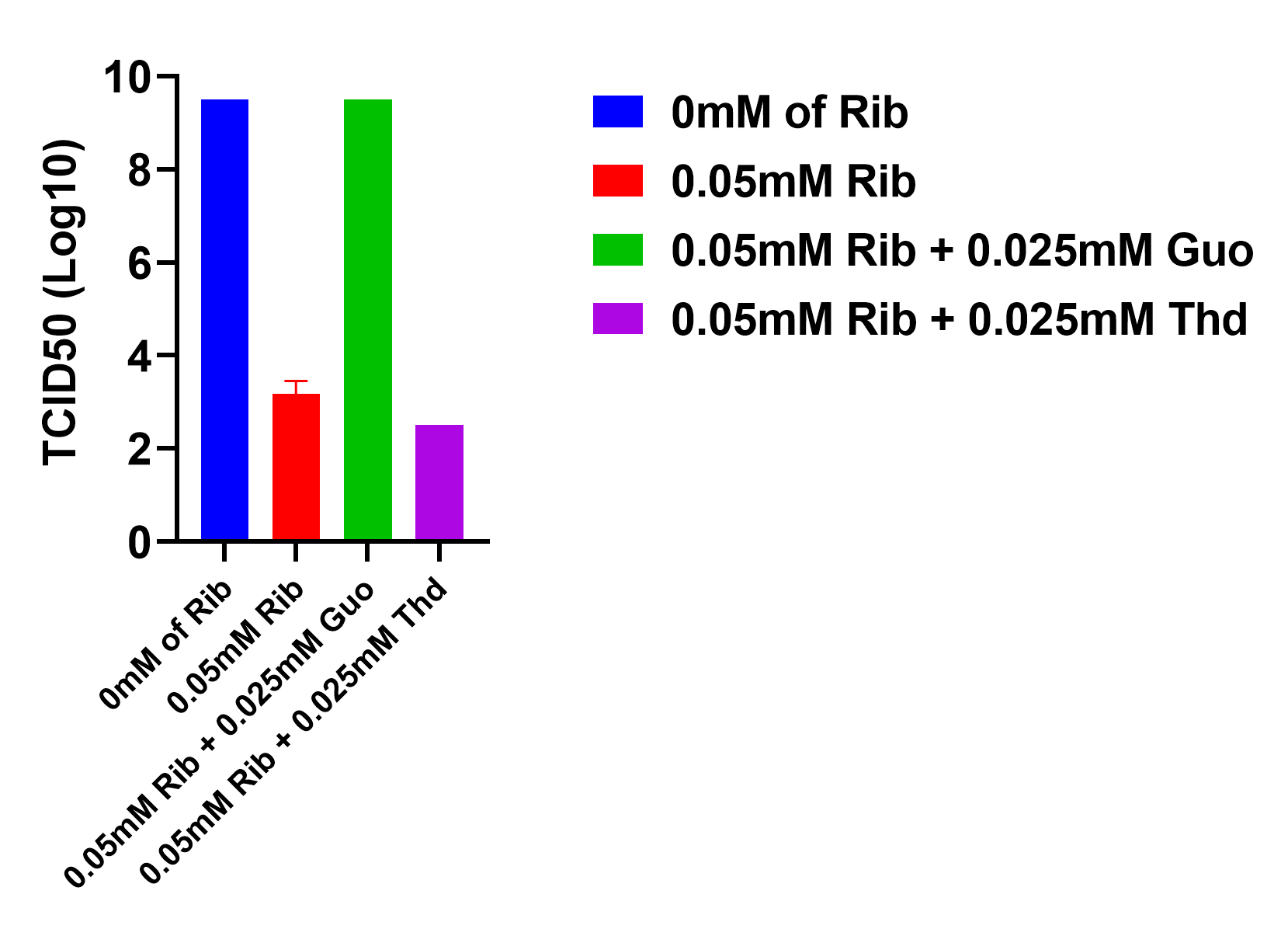


**Fig S2.** The effect of indicated concentration of ribavirin (Rib) in presence or absence of Guanosine (Guo) Supplementation on the replication of FVSKG2 in CEFs. Virus titres are presented as 50% tissue culture infective dose (TCID50/ml, log10) per milliliter (y axis). Errors bars denote mean ± standard error of mean (SEM). Thymidine (ThD) used as the control.
