# Supplementary material for: Ribavirin inhibits the replication of infectious bursal disease virus predominantly through depletion of cellular guanosine pool
Source: Front Vet Sci. 2023 Jul 31;10:1192583. doi: 10.3389/fvets.2023.1192583 (PMC10433155; doi:10.3389/fvets.2023.1192583)
Supplement: Supplementary file 4 [file Data_Sheet_2.pdf]

ClustalW sequence alignment of partial RNA Dependent RNA Polymerase (RdRp) of IBD virus serially passaged in presence of 0mM Ribavirin (FVSKG2_RDRP-1) and 0.025mM ribavirin (FVSKG2_0.025mM) at 5th passage.

```
FVSKG2_RDRP-1      GCTCACCAAGACCCGGAACATATGGTCAGCTCCATCCCCAACACACCTCATGATCTCCAT    60
FVSKG2_0.025mM     GCTCACCAAGACCCGGAACATATGGTCAGCTCCATCCCCAACACACCTCATGATCTCCAT    60
                   ************************************************************

FVSKG2_RDRP-1      GATCACCTGGCCCGTGATGTCCAACAGCCCAAATAACGTGTTGAACATTGAAGGGTGTCC    120
FVSKG2_0.025mM     GATCACCTGGCCCGTGATGTCCAACAGCCCAAATAACGTGTTGAACATTGAAGGGTGTCC    120
                   ************************************************************

FVSKG2_RDRP-1      ATCACTCTACAAATTCAACCCGTTCAGAGGAGGGTTGAACAGGATCGTCGAGTGGATATT    180
FVSKG2_0.025mM     ATCACTCTACAAATTCAACCCGTTCAGAGGAGGGTTGAACAGGATCGTCGAGTGGATATT    180
                   ************************************************************

FVSKG2_RDRP-1      GGCCCCGGAAGAACCCAAGGCTCTTGTATATGCGGACAACATATACATTGTCCACTCAAA    240
FVSKG2_0.025mM     GGCCCCGGAAGAACCCAAGGCTCTTGTATATGCGGACAACATATACATTGTCCACTCAAA    240
                   ************************************************************

FVSKG2_RDRP-1      CACGTGGTACTCAATTGACCTAGAGAAGGGTGAGGCAAACTGCACTCGCCAACACATGCA    300
FVSKG2_0.025mM     CACGTGGTACTCAATTGACCTAGAGAAGGGTGAGGCAAACTGCACTCGCCAACACATGCA    300
                   ************************************************************

FVSKG2_RDRP-1      AGCCGCAATGTACTACATACTCACCAGAGGGTGGTCAGACAACGGCGACCCAATGTTCAA    360
FVSKG2_0.025mM     AGCCGCAATGTACTACATACTCACCAGAGGGTGGTCAGACAACGGCGACCCAATGTTCAA    360
                   ************************************************************

FVSKG2_RDRP-1      TCAAACATGGGCCACCTTTGCCATGAACATTGCCCCTGCTCTAGTGGTGGACTCATCGTG    420
FVSKG2_0.025mM     TCAAACATGGGCCACCTTTGCCATGAACATTGCCCCTGCTCTAGTGGTGGACTCATCGTG    420
                   ************************************************************

FVSKG2_RDRP-1      CCTGATAATGAACCTGCAAATTAAGACCTATGGTCAAGGCAGCGGGAATGCAGCCACGTT    480
FVSKG2_0.025mM     CCTGATAATGAACCTGCAAATTAAGACCTATGGTCAAGGCAGCGGGAATGCAGCCACGTT    480
                   ************************************************************

FVSKG2_RDRP-1      CATCAACAACCACCTCTTGAGCACGCTAGTGCTTGACCAGTGGAACTTGATGAGACAGCC    540
FVSKG2_0.025mM     CATCAACAACCACCTCTTGAGCACGCTAGTGCTTGACCAGTGGAACTTGATGAGACAGCC    540
                   ************************************************************
```

```
FVSKG2_RDRP-1       CAGACCAGACAGCGAGGAGTTCAAATCAATTGAGGACAAGCTAGGTATCAACTTTAAGAT    600
FVSKG2_0.025mM      CAGACCAGACAGCGAGGAGTTCAAATCAATTGAGGACAAGCTAGGTATCAACTTTAAGAT    600
                    ************************************************************

FVSKG2_RDRP-1       TGAGAGGTCCATTGATGATATCAGGGGCAAGCTGAGACAGCTTGTCCTCCTTGCACAACC    660
FVSKG2_0.025mM      TGAGAGGTCCATTGATGATATCAGGGGCAAGCTGAGACAGCTTGTCCTCCTTGCACAACC    660
                    ************************************************************

FVSKG2_RDRP-1       AGGGTACCTGAGTGGGGGGGTTGAACCAGAACAATCCAGCCCAACTGTTGAGCTTGACCT    720
FVSKG2_0.025mM      AGGGTACCTGAGTGGGGGGGTTGAACCAGAACAATCCAGCCCAACTGTTGAGCTTGACCT    720
                    ************************************************************

FVSKG2_RDRP-1       ACTAGGGTGGTCAGCTACATACAGCAAAGATCTCGGGATCTATGTGCCGGTGCTTGACAA    780
FVSKG2_0.025mM      ACTAGGGTGGTCAGCTACATACAGCAAAGATCTCGGGATCTATGTGCCGGTGCTTGACAA    780
                    ************************************************************

FVSKG2_RDRP-1       GGAACGCCTATTTTGTTCTGCTGCGTATCCCAAGGGAGTAGAGAACAAGAGTCTCAAGTC    840
FVSKG2_0.025mM      GGAACGCCTATTTTGTTCTGCTGCGTATCCCAAGGGAGTAGAGAACAAGAGTCTCAAGTC    840
                    ************************************************************

FVSKG2_RDRP-1       CAAAGTCGGGATCGAGCAGGC     861
FVSKG2_0.025mM      CAAAGTCGGGATCGAGCAGGC     861
                    *********************
```

ClustalW sequence alignment of partial sequence of VP2 of IBD virus serially passaged in presence of 0mM Ribavirin (FVSKG2_VP2) and 0.025mM ribavirin (FVSKG2_0.025mM) at 5th passage.

```
FVSKG2_VP2          AGAATTGTTCGgTTCATASGGAGCYTTCTGATGCCAACAACCGGACYGGCGTCCATTCCG    60
FVSKG2_0.025mM      AGAATTGTTCGgTTCATASGGAGCYTTCTGATGCCAACAACCGGACYGGCGTCCATTCCG    60
                    ************************************************************

FVSKG2_VP2          GACGACACCSTGGAGAAGCACACTCTCAGGTCAGAGACCTCGACCTACAATTTGACTGTG    120
FVSKG2_0.025mM      GACGACACCSTGGAGAAGCACACTCTCAGGTCAGAGACCTCGACCTACAATTTGACTGTG    120
                    ************************************************************

FVSKG2_VP2          GGGGACACAGGGTCAGGGCTAATTGTCTTTTTCCCTGGATTCCCTGGCTCAATTGTGGGT    180
FVSKG2_0.025mM      GGGGACACAGGGTCAGGGCTAATTGTCTTTTTCCCTGGATTCCCTGGCTCAATTGTGGGT    180
                    ************************************************************

FVSKG2_VP2          GCTCACTACACACTGCAGAGCAATGGGAACTACAAGTTCGATCAGATGCTCCTGACTGCC    240
FVSKG2_0.025mM      GCTCACTACACACTGCAGAGCAATGGGAACTACAAGTTCGATCAGATGCTCCTGACTGCC    240
                    ************************************************************

FVSKG2_VP2          CAGAACCTACCGGCCAGTTACAACTACTGCAGGCTAGTGAGTCGGAGTCTCACAGTGAGG    300
FVSKG2_0.025mM      CAGAACCTACCGGCCAGTTACAACTACTGCAGGCTAGTGAGTCGGAGTCTCACAGTGAGG    300
                    ************************************************************

FVSKG2_VP2          TCAAGCACACTTCCTGGTGGCGTTTATGCACTAAACGGCACCATAAACGCCGTGACCTTC    360
FVSKG2_0.025mM      TCAAGCACACTTCCTGGTGGCGTTTATGCACTAAACGGCACCATAAACGCCGTGACCTTC    360
                    ************************************************************

FVSKG2_VP2          CAAGGAAGCCTGAGTGAACTGACAGATGTTAGCTACAATGGGTTGATGTCTGCAACAGCC    420
FVSKG2_0.025mM      CAAGGAAGCCTGAGTGAACTGACAGATGTTAGCTACAATGGGTTGATGTCTGCAACAGCC    420
                    ************************************************************

FVSKG2_VP2          AACATCAACGACAAAATTGGGAACGTCCTAGTAGGGGAAGGGGTCACCGTCCTCAGCTTA    480
FVSKG2_0.025mM      AACATCAACGACAAAATTGGGAACGTCCTAGTAGGGGAAGGGGTCACCGTCCTCAGCTTA    480
                    ************************************************************

FVSKG2_VP2          CCCACATCATATGATCTTGGGTATGTGAGGCTTGGTGACCCCATTCCCGCAATAGGGCTT    540
FVSKG2_0.025mM      CCCACATCATATGATCTTGGGTATGTGAGGCTTGGTGACCCCATTCCCGCAATAGGGCTT    540
                    ************************************************************
```

```
FVSKG2_VP2          GACCCAAAAATGGTAGCCACATGTGACAGCAGTGACAGGCCCAGAGTCTACACCATAACT    600
FVSKG2_0.025mM      GACCCAAAAATGGTAGCCACATGTGACAGCAGTGACAGGCCCAGAGTCTACACCATAACT    600
                    ************************************************************

FVSKG2_VP2          GCAGCCRATGATTACCAATTCTCATCACAGTACCAACCAGGTGGGGTAACAATCACACTG    660
FVSKG2_0.025mM      GCAGCCRATGATTACCAATTCTCATCACAGTACCAACCAGGTGGGGTAACAATCACACTG    660
                    ************************************************************

FVSKG2_VP2          TTCTCAGCCAACATTGATGCCATCACAAGCCTCAGCGTTGGGGGAGAGCTCGTGTTTCAA    720
FVSKG2_0.025mM      TTCTCAGCCAACATTGATGCCATCACAAGCCTCAGCGTTGGGGGAGAGCTCGTGTTTCAA    720
                    ************************************************************

FVSKG2_VP2          ACAAGCGTCCACGGCCTTGTACTGGGCGCCACCATCTACCTCATAGGCTTTGATGGGACA    780
FVSKG2_0.025mM      ACAAGCGTCCACGGCCTTGTACTGGGCGCCACCATCTACCTCATAGGCTTTGATGGGACA    780
                    ************************************************************

FVSKG2_VP2          ACGGTAATCACCARGGCTGTGGCCGCAAACAATGGGCTGACGACCGGCACCGACAACCTT    840
FVSKG2_0.025mM      ACGGTAATCACCARGGCTGTGGCCGCAAACAATGGGCTGACGACCGGCACCGACAACCTT    840
                    ************************************************************

FVSKG2_VP2          ATGCCATTCAATCTTGTGATCCACACGAGTA   871
FVSKG2_0.025mM      ATGCCATTCAATCTTGTGATCCACACGAGTA   871
                    *******************************
```
